# Analysis of Prenatal Exposure to Opioid Analgesics and Scholastic Skills in Children in Fifth Grade in Norway

**DOI:** 10.1001/jamanetworkopen.2022.22425

**Published:** 2022-07-19

**Authors:** Johanne Naper Trønnes, Angela Lupattelli, Eivind Ystrom, Hedvig Nordeng

**Affiliations:** 1 PharmacoEpidemiology and Drug Safety Research Group, Department of Pharmacy, and PharmaTox Strategic Research Initiative, Faculty of Mathematics and Natural Sciences, University of Oslo, Oslo, Norway; 2 Department of Mental Disorders, Norwegian Institute of Public Health, Oslo, Norway; 3 PROMENTA Research Center, Department of Psychology, University of Oslo, Oslo, Norway; 4 Department of Child Health and Development, Norwegian Institute of Public Health, Oslo, Norway

## Abstract

**Importance:**

Few studies have examined the neurodevelopmental consequences of prenatal exposure to opioid analgesics. Therefore, it is necessary to gain knowledge to inform clinical decisions for pregnant women with moderate to severe pain.

**Objective:**

To investigate fifth-grade scholastic skills in children with prenatal exposure to opioid analgesics.

**Design, Setting, and Participants:**

A cohort study was conducted based on data from the Norwegian Mother, Father, and Child Cohort (1999-2008). These data were linked to the Medical Birth Registry of Norway, and data from Statistics Norway included 64 256 live-born singletons, born to 54 568 mothers who reported pain during pregnancy, were analyzed. The study was conducted from July 1 to December 15, 2021.

**Exposures:**

Self-reported exposure to opioid analgesics during pregnancy, characterized in terms of any exposure, the exposure timing, and the exposure duration.

**Main Outcomes and Measures:**

Scores from 3 national tests for children in fifth grade. The tests measured scholastic skills in literacy, numeracy, and the English language. Test scores were standardized to *z* scores. Differences in z scores were compared between children of mothers exposed to opioid analgesics during pregnancy and children of mothers with only prepregnancy opioid exposure.

**Results:**

Of the 64 256 children included, 32 521 were boys (50.6%), and 1483 children (2.3%) were exposed to an opioid analgesic at least once during gestation. All test scores were similar between children with any exposure to opioid analgesics in utero and children with only prepregnancy exposure. Children exposed in the first trimester and those exposed in two or three 4-week intervals during pregnancy scored lower than children of mothers with only prepregnancy exposures on tests in literacy (weighted β [wβ], −0.13; 95% CI, −0.25 to −0.01 and wβ, −0.19; 95% CI, −0.35 to −0.04) and numeracy (wβ, −0.14; 95% CI, −0.25 to −0.04 and wβ, −0.19; 95% CI, −0.34 to −0.05). These differences were small and may not be clinically relevant.

**Conclusions and Relevance:**

In this large birth cohort, prenatal exposure to opioid analgesics had no substantial negative association with fifth-grade scholastic skills. However, adequate pain management in pregnancy may be addressed on an individual patient level, bearing in mind the benefits and risks of different analgesic therapies.

## Key Points

**Question:**

Is there an association between prenatal exposure to opioid analgesics and fifth-grade scholastic skills?

**Findings:**

In this cohort study of 64 256 children, exposure to opioid analgesics in the first trimester or during two to three 4-week intervals during pregnancy was associated with lower scores in literacy and numeracy tests, compared with only prepregnancy exposure. However, the differences may not be clinically relevant.

**Meaning:**

The findings of this study suggest that there is no association between prenatal exposure to opioid analgesics and fifth-grade scholastic skills.

## Teaser

This cohort study examines the scholastic abilities of children in the fifth grade who were exposed prenatally to opioid analgesics.

## Introduction

Prescription opioid analgesics are used by 3% to 22% of individuals who are pregnant.^1,2,3,4^ Animal research has shown that prenatal exposure to opioids alters brain structures and functions; thus, opioids might interfere with fetal neurodevelopment.^5,6,7^ In light of the ongoing opioid epidemic,^8^ a major concern has been the lack of knowledge about the neurodevelopmental consequences of prenatal exposure to opioid analgesics.

To our knowledge, only 4 previous studies have examined the outcomes of prenatal exposure to opioid analgesics and child neurodevelopment.^9,10,11,12,13^ Three of those studies^11,12,13^ were based on a large Norwegian birth cohort. Skovlund et al^12,13^ reported that prenatal analgesic opioid exposure was not associated with impaired language competence or communication skills in preschool children. However, prenatal exposures for 5 or more weeks slightly increased the risk of an attention-deficit/hyperactivity disorder diagnosis, compared with shorter exposures (hazard ratio, 1.60; 95% CI, 1.04-2.47).^11^ Similarly, Wen et al^10^ reported that prenatal exposures for more than 14 days or exposures to high cumulative opioid doses increased the risk of neurodevelopmental disorders (hazard ratio range, 1.22-1.70), compared with no exposure.

Scholastic skills are important indicators of cognitive function, but they are infrequently assessed in perinatal pharmacoepidemiologic studies.^14,15,16^ Scholastic skills, including reading and mathematics abilities, depend on cognitive processes related to executive function and working memory.^17^ Thus, scholastic skills can estimate future academic achievement, career aptitudes, and socioeconomic status.^18,19^ We aimed to investigate whether fifth-grade scholastic skills are associated with prenatal exposure to opioid analgesics, based on any exposure, exposure timing, and exposure durations, with adjustments for important confounders.

## Methods

### Data Sources and Study Sample

Data for this cohort study were retrieved from the Norwegian Mother, Father, and Child Cohort Study (MoBa), the Medical Birth Registry of Norway, and Statistics Norway. Data were linked via the unique personal identification number given to all residents of Norway.

MoBa was a population-based pregnancy cohort study conducted by the Norwegian Institute of Public Health.^20^ Participants were recruited throughout Norway between 1999 and 2008. In 41% of the pregnancies, women consented to cohort participation. The cohort includes 114 500 children, 95 200 mothers, and 75 200 fathers. Mothers were followed up with paper-based questionnaires during pregnancy and after delivery. The present study is based on version 12 of the quality-assured data files released for research in 2019, with the present study conducted from July 1 to December 15, 2021. The establishment of MoBa and the initial data collection were based on a license from the Norwegian Data Protection Agency and approved by the Regional Committees for Medical and Health Research Ethics. The MoBa cohort is currently based on regulations related to the Norwegian Health Registry Act. The present study was approved by the Regional Committees for Medical and Health Research Ethics. Written informed consent was obtained from all participants. No financial compensation was provided. The Strengthening the Reporting of Observational Studies in Epidemiology (STROBE) reporting guideline was followed.

The Medical Birth Registry of Norway is a national health registry that has stored information on all births in Norway, starting in 1967.^21^ Statistics Norway contains information from public registries.^22^ For the present study, we acquired data on parental educational level, family income, and children’s school test results.

We included all mother-child dyads of singleton pregnancies that were enrolled in the MoBa study between 2002 and 2008 and were recorded in the Medical Birth Registry of Norway. To account for confounding by indication, we restricted the study sample to women who reported indications for opioid analgesia during pregnancy (ie, pain conditions) (eMethods, eTable 1 in the Supplement). Other inclusion and exclusion criteria are presented in the Figure.

**Figure.**
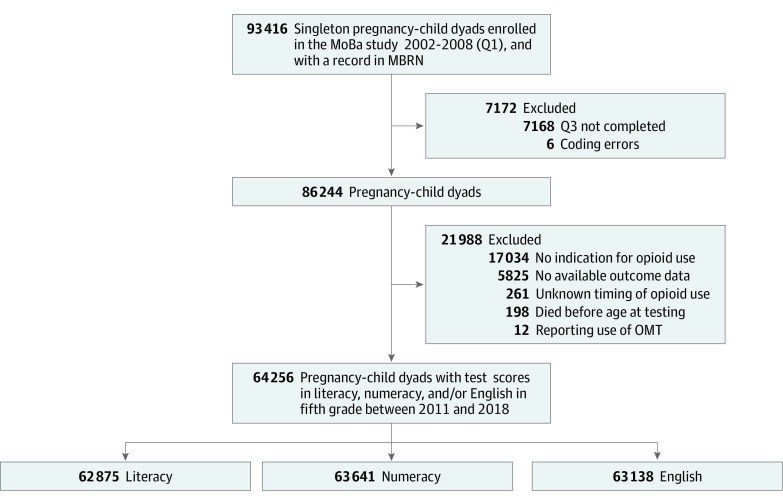
Flowchart to Achieve Study Samples Norwegian Mother, Father, and Child Cohort Study (MoBa) children born in 1999-2001 were not included in the study owing to lack of consent, because they became age 18 years before the follow-up was completed in 2018. Conditions of exclusion can overlap. MBRN indicates Medical Birth Registry of Norway; OMT, opioid maintenance treatment (ie, women reporting use of drugs with Anatomical Therapeutic Chemical Classification code N07BC); Q1, first MoBa questionnaire; and Q3, third MoBa questionnaire.

### Exposure

Medication use was self-reported by the mothers in 2 prenatal and 1 postpartum questionnaire. The mothers also indicated whether they had experienced any illnesses among a list of short- and long-term illnesses. In addition, they reported any medication use and specified the timing, starting at 6 months before pregnancy and continuing throughout the pregnancy, based on 4-week intervals (eg, gestational weeks 0-4, 5-8, or 9-12).

Exposure was defined as the mother’s use of analgesic opioids (N02A in the World Health Organization’s Anatomical Therapeutic Chemical Classification System^23^). We defined any exposure to opioid analgesics during pregnancy as use initiated during pregnancy and use that had started before pregnancy and was continued during pregnancy. We also defined opioid exposures based on timing and duration. Timing was categorized into trimesters (first trimester [0-12 weeks of gestation], second trimester [13-28 weeks of gestation], and third trimester [29 weeks to delivery]). The duration of opioid use was indicated as the number of 4-week intervals that opioids were used during pregnancy (categorized as one, two to three, or four or more 4-week intervals). However, use in an interval did not necessarily mean consecutive use during that period.

To evaluate the outcomes of opioid exposure among all pregnant women with pain ailments, we defined 2 mutually exclusive reference groups. Our main reference group comprised children of mothers who used opioid analgesics only before pregnancy (prepregnancy exposure). In a subanalysis, we used a reference group that included children of mothers who did not report opioid analgesics use before or during pregnancy (unexposed).

### Outcomes

The outcomes were the scores from 3 national standardized tests on literacy, numeracy, and the English language. These tests were mandatory for children in the fifth grade (age 10-11 years); only children with special educational or special language training needs were exempted from a test.^24^ We had access to test results for the complete population of children in the fifth grade in the study period. The test scores were standardized as z scores over the total population of children taking the tests in each subject and for each test year. A z score of −1 indicated a test score of 1 SD lower than the population mean. More information is provided in the eMethods in the Supplement, and the distribution of raw test scores is shown in eFigure 1 in the Supplement. Raw test scores were compared between the MoBa participants and the total population of children taking the tests (eTable 2 and eTable 3 in the Supplement).

### Covariates

Potential confounders and risk factors for the outcome were identified a priori based on subject knowledge and directed acyclic graphs (eFigure 2 in the Supplement).^25,26,27,28^ The sources of different covariates are reported in eTable 4 in the Supplement. The following covariates were included in our main analysis and characterized as described in Table 1: maternal age at delivery, marital status, parity, maternal and paternal educational levels, family income-to-needs ratio (1 year before childbirth), prepregnancy body mass index, chronic maternal diseases, smoking habits before pregnancy, alcohol use, use of comedications, symptoms of anxiety and depression^29^ (measured on the first MoBa questionnaire), time of year the baby was born (before/after summer), and paternal age.

**Table 1.** Characteristics of the Study Sample According to Prenatal Opioid Exposure Status (N = 64 256)

Characteristic	Exposure status, No. (%)		
	Exposed (n = 1483)	Prepregnancy exposed only (n = 731)	Unexposed (n = 62 042)
**Maternal characteristics**			
Age at delivery, y			
<25	155 (10.4)	107 (14.6)	7075 (11.4)
25-29	473 (31.9)	241 (32.9)	21 041 (33.9)
30-34	554 (37.4)	271 (37.1)	23 794 (38.4)
≥35	301 (20.3)	112 (15.4)	10 132 (16.3)
Marital status			
Married/cohabitant	1410 (95.1)	697 (95.4)	59 865 (96.5)
Other	66 (4.4)	30 (4.1)	1876 (3.0)
Missing	7 (0.5)	4 (0.5)	301 (0.5)
Parity			
Primiparous	607 (40.9)	400 (54.7)	27 878 (44.9)
Multiparous	876 (59.1)	331 (45.3)	34 164 (55.1)
Educational level^a^			
10-y Primary school or less	168 (11.3)	81 (11.1)	4955 (8.0)
Secondary/vocational school	476 (32.1)	242 (33.1)	17 749 (28.6)
College or advanced degree	835 (56.3)	405 (55.4)	38 912 (62.7)
Missing	4 (0.3)	3 (0.4)	426 (0.7)
Family income, ITNR^b^			
<2	590 (39.8)	290 (39.7)	22 069 (35.6)
2-3	689 (46.5)	313 (42.8)	28 876 (46.5)
≥3	194 (13.1)	123 (16.8)	10 469 (16.9)
Missing	10 (0.6)	5 (0.7)	628 (1.0)
Prepregnancy BMI, mean (SD)	25.1 (4.9)	24.8 (4.7)	24.1 (4.3)
Missing	36 (2.4)	18 (2.5)	1535 (2.5)
Smoking^c^			
No	1033 (69.6)	506 (69.2)	46 083 (74.3)
Yes	213 (14.4)	111 (15.2)	5795 (9.3)
Missing	237 (16.0)	114 (15.6)	10 164 (16.4)
Alcohol^d^			
No	1261 (85.0)	648 (88.6)	54 775 (88.3)
Yes	52 (3.5)	19 (2.6)	1449 (2.3)
Missing	170 (11.5)	64 (8.8)	5818 (9.4)
Symptoms of anxiety/depression score, mean (SD)^e^	1.4 (0.5)	1.4 (0.4)	1.3 (0.4)
Missing	63 (4.2)	24 (3.3)	2067 (3.3)
Maternal chronic disease^f^			
No	1188 (80.1)	585 (80.0)	53 917 (86.9)
Yes	295 (19.9)	146 (20.0)	8125 (13.1)
Use of comedication^d^			
Acetaminophen	728 (49.1)	238 (32.6)	17 811 (28.7)
Triptans	80 (5.4)	5 (0.7)	475 (0.8)
NSAIDs	210 (14.2)	42 (5.8)	2783 (4.5)
Antidepressants	39 (2.6)	11 (1.5)	588 (1.0)
Benzodiazepines and benzodiazepinelike drugs	46 (3.1)	5 (0.7)	252 (0.4)
Antiepileptic	10 (0.7)	4 (0.6)	186 (0.3)
Antipsychotic	22 (1.5)	8 (1.1)	368 (0.6)
Pain types reported during pregnancy, No.			
1	224 (15.1)	86 (11.8)	16 322 (26.3)
2-3	502 (33.9)	286 (39.1)	27 530 (44.4)
≥4	757 (51.0)	359 (49.1)	18 190 (29.3)
**Child characteristics**			
Sex			
Boys	740 (49.9)	359 (49.1)	31 422 (50.6)
Girls	743 (50.1)	372 (50.9)	30 620 (49.4)
Preterm (<37 wk)	99 (6.7)	35 (4.8)	2644 (4.3)
Missing	5 (0.3)	3 (0.4)	248 (0.4)
Time of year the baby was born			
January-June	775 (52.3)	381 (52.1)	31 592 (50.9)
July-December	708 (47.7)	350 (47.9)	30 450 (49.1)
**Paternal characteristics**			
Age, y			
25-29	398 (26.8)	231 (31.6)	17 516 (28.2)
30-34	568 (38.3)	263 (35.9)	24 159 (38.9)
≥35	514 (34.7)	236 (32.3)	20 235 (32.6)
Missing	3 (0.2)	1 (0.2)	132 (0.2)
Educational level^a^			
10 y Primary school or less	199 (13.4)	116 (15.9)	6813 (11.0)
Secondary/vocational school	688 (46.4)	320 (43.8)	26 597 (42.9)
College or advanced degree	583 (39.3)	285 (38.9)	27 831 (44.8)
Missing	13 (0.9)	10 (1.4)	801 (1.3)

Abbreviations: BMI, body mass index (calculated as weight in kilograms divided by height in meters squared); ITNR, income to needs ratio; NSAIDs, nonsteroidal anti-inflammatory drugs.

^a^ Educational level was reported in the child’s birth year.

^b^ Family income was assessed by ITNR (EU-60 [European standard on how to calculate income to needs ratio]) reported in the year before childbirth.

^c^ Smoking status was reported at the start of pregnancy.

^d^ Measured in the first Mother, Father, and Child Cohort Study (MoBa) questionnaire.

^e^ Symptoms of anxiety and depression were measured by a short version of the Hopkins Symptom Checklist in the first MoBa questionnaire.

^f^ Maternal chronic disease include asthma, diabetes, hypertension, other heart disease, epilepsy, thyroid disorder, or arthritis reported 6 months before pregnancy.

### Statistical Analysis

To account for the measured confounders and risk factors, we implemented propensity score (PS)^30^ methods with an inverse probability of treatment weights. Each PS was derived with a logistic regression model.^31^ In analyzing timing, the PS was estimated as the probability of opioid analgesic exposure in the first trimester, second trimester, and third trimester, compared with only prepregnancy exposure. In analyzing duration, the PS was estimated as the probability of opioid analgesic exposure in 1 interval, 2 to 3 intervals, and 4 or more intervals, compared with only prepregnancy exposure. Then, we derived the respective weights. The covariates were balanced, based on the standardized mean differences, and a standardized mean difference greater than 0.15 indicated an imbalance (eTable 5 in the Supplement).^32^ We fit generalized linear models with robust SEs to obtain crude and weighted standardized mean differences in test scores with 95% CIs. We used 95% CIs to describe the precision of our estimates, corresponding to an α level of .05 for a 2-sided test. All statistical analyses were performed with Stata MP, version 16 (StataCorp LLC).

Up to 29.3% of the included pregnancies had missing values for at least 1 of the important confounders. The variables with the highest proportion of missing values were smoking status (16.4%), alcohol use (9.4%), and depressive and anxiety symptoms (3.4%). Assuming that data were missing at random, we imputed incomplete data by performing multiple imputations with chained equations (30 replications).^33,34,35^ Data were imputed separately for the different tests (literacy, numeracy, and English language). The PS and subsequent weights were estimated in each imputed data set. Then, the PSs were applied to estimate individual exposure outcomes associated with literacy, numeracy, and the English language. The individual exposure estimates were combined to produce an overall exposure estimate.^36,37^

We did several sensitivity analyses. First, we conducted an analysis with unexposed children as the reference group. Second, we performed sex-stratified analyses to investigate whether associations between opioid exposure and scholastic skills were similar among boys and girls. Third, we conducted a complete case analysis and compared the results with the imputed data set results. Fourth, we performed an analysis in which we compared children exposed for one 4-week interval with those exposed for two or more 4-week intervals during pregnancy. Fifth, we repeated our main analysis with an alternative model specification (eTable 6 in the Supplement). Additional sensitivity analyses are described in the eMethods in the Supplement.

## Results

The study included 64 256 children of 54 568 mothers (mean [SD] maternal age, 30.5 [4.5] years). Of these children, 32 521 (50.6%) were boys and 31 735 (49.4%) were girls. Opioid analgesic use was reported in 2.3% of pregnancies (n = 1483). The dominating substance was codeine combined with acetaminophen, reported by 90.5% of the exposed women (eTable 7 in the Supplement). Most women reported short-term use (937 of 1483 [63.2%]); ie, opioids were used in one 4-week interval during pregnancy. Mothers of exposed children were slightly older, more likely to have previous children, and more likely to report alcohol and comedication use, compared with mothers with prepregnancy analgesic opioid exposure (Table 1).

### Scholastic Skills

The Figure shows the number of children who participated in each test; most (61814 [96.2%]) participated in all 3 tests. Among our study sample of 64 256 children, very few (literacy, 0.8%; numeracy, 0.3%; and English language, 0.9%) were exempted (eFigure 3 in the Supplement). Among the exempted children, between 2.7% and 3.6% were born to mothers who used opioid analgesics during pregnancy. Across the tests, approximately 13% of children scored 1 SD below the population mean on the tests. Children with any opioid analgesic exposure during pregnancy did not score lower on tests in literacy, numeracy, or the English language, compared with children of mothers with only prepregnancy opioid exposure (Table 2).

**Table 2.** Association Between Timing and Duration of Prenatal Exposure to Opioid Analgesics and Scholastic Skills Among Children in Fifth Grade Compared With Children of Mothers With Prepregnancy Exposure

Characteristic	No.	*z* score, mean (SD)	β (95% CI)^a^	
			Crude	Weighted
**Literacy**				
Prepregnancy exposed only	721	0.21 (1.0)	1 [Reference]	1 [Reference]
Exposed	1445	0.15 (1.0)	0.06 (−0.15 to 0.03)	−0.06 (−0.16 to 0.04)
By timing of exposure, trimester				
First	671	0.09 (1.0)	−0.12 (−0.23 to −0.02)	−0.13 (−0.25 to −0.01)
Second	783	0.17 (1.0)	−0.04 (−0.14 to 0.05)	−0.05 (−0.16 to 0.05)
Third	486	0.18 (1.0)	−0.04 (−0.14 to 0.07)	−0.03 (−0.15 to 0.09)
By duration of exposure, intervals^b^				
1	917	0.18 (1.0)	−0.03 (−0.13 to 0.06)	−0.03 (−0.13 to 0.07)
2-3	313	0.09 (1.0)	−0.13 (−0.26 to −0.00)	−0.19 (−0.35 to −0 04)
≥4	215	0.12 (1.0)	−0.09 (−0.24 to 0.06)	−0.02 (−0.21 to 0.16)
**Numeracy**				
Prepregnancy exposed only	722	0.21 (1.0)	1 [Reference]	1 [Reference]
Exposed	1469	0.11 (1.0)	−0.09 (−0.18 to −0.01)	−0.08 (−0.17 to 0.01)
By timing of exposure, trimester				
First	677	0.05 (1.0)	−0.16 (−0.26 to −0.06)	−0.14 (−0.25 to −0.04)
Second	800	0.15 (1.0)	−0.06 (−0.15 to 0.04)	−0.06 (−0.16 to 0.04)
Third	492	0.13 (1.0)	−0.08 (−0.18 to 0.04)	−0.05 (−0.18 to 0.07)
By duration of exposure, intervals^b^				
1	929	0.14 (1.0)	−0.07 (−0.16 to 0.03)	−0.06 (−0.16 to 0.03)
2-3	322	0.05 (1.0)	−0.16 (−0.29 to −0.03)	−0.19 (−0.34 to −0.05)
≥4	218	0.10 (1.0)	−0.10 (−0.25 to 0.04)	−0.05 (−0.25 to 0.14)
**English**				
Prepregnancy exposed only	718	0.07 (1.0)	1 [Reference]	1 [Reference]
Exposed	1444	0.08 (1.0)	−0.02 (−0.11 to 0.07)	−0.04 (−0.13 to 0.06)
By timing of exposure, trimester				
First	672	0.01 (1.0)	−0.06 (−0.17 to 0.04)	−0.06 (−0.17 to 0.06)
Second	780	0.08 (1.0)	0.01 (−0.09 to 0.11)	−0.02 (−0.13 to 0.09)
Third	484	0.08 (1.0)	0.01 (−0.11 to 0.12)	−0.02 (−0.15 to 0.10)
By duration of exposure, intervals^b^				
1	917	0.05 (1.0)	−0.02 (−0.12 to 0.08)	−0.03 (−0.13 to 0.06)
2-3	313	0.01 (1.0)	−0.07 (−0.20 to 0.07)	−0.11 (−0.26 to 0.05)
≥4	214	0.11 (1.0)	0.04 (−0.12 to 0.19)	0.12 (−0.10 to 0.35)

^a^ β, standardized mean difference in test scores.

^b^ One interval corresponds to a 4-week period, but not necessarily consecutive use in that period.

In the analyses of exposure timing, children exposed to opioid analgesics in the first trimester scored lower on tests in literacy (weighted β [wβ], −0.13; 95% CI, −0.25 to −0.01) and numeracy (wβ, −0.14; 95% CI, −0.25 to −0.04) compared with children of mothers with only prepregnancy exposure. Children exposed in the second or third trimester did not score significantly lower in any subject, although they had trends of lower scores, compared with children of mothers with only prepregnancy exposure.

In the analyses of duration, children exposed in two to three 4-week intervals during pregnancy scored lower on tests in literacy (wβ, −0.19; 95% CI, −0.35 to −0.04) and numeracy (wβ, −0.19; 95% CI, −0.34 to −0.05), compared with children of mothers with only prepregnancy exposure. No other exposure durations were associated with lower test scores in literacy, numeracy, or the English language.

### Sensitivity Analyses

In crude analyses with unexposed children as the reference group, we observed similar patterns of associations to those observed in the main analyses. However, after adjustments, all 95% CIs included the null (Table 3). In analyses stratified by sex, we found no difference between boys and girls; the point estimates were of similar magnitude and 95% CIs were overlapping (eTable 8 in the Supplement). Results from the complete case analyses did not differ substantially from the results from the main analysis. Results from the remaining sensitivity analyses are described in the eResults and eTable 9 in the Supplement.

**Table 3.** Timing and Duration of Prenatal Exposure to Opioid Analgesics and Scholastic Skills Among Children in Fifth Grade Compared With Unexposed Children

Characteristic	No.	*z* Score, mean (SD)	β (95% CI)^a^	
			Crude	Weighted
**Literacy**				
Unexposed	60 709	0.21 (1.0)	1 [Reference]	1 [Reference]
Exposed	1445	0.15 (1.0)	−0.05 (−0.10 to 0.00)	0.01 (−0.06 to 0.04)
By timing of exposure, trimester				
First	671	0.09 (1.0)	−0.12 (−0.19 to −0.04)	−0.06 (−0.14 to 0.01)
Second	783	0.17 (1.0)	−0.04 (−0.11 to 0.03)	0.01 (−0.06 to 0.08)
Third	486	0.18 (1.0)	−0.03 (−0.11 to 0.06)	0.00 (−0.09 to 0.08)
By duration of exposure, intervals^b^				
1	917	0.18 (1.0)	−0.02 (−0.09 to 0.04)	0.02 (−0.04 to 0.08)
2-3	313	0.09 (1.0)	−0.13 (−0.23 to −0.02)	−0.09 (−0.20 to 0.02)
≥4	215	0.12 (1.0)	−0.08 (−0.21 to 0.04)	0.01 (−0.11 to 0.14)
**Numeracy**				
Unexposed	61 450	0.20 (1.0)	1 [Reference]	1 [Reference]
Exposed	1469	0.11 (0.1)	−0.09 (−0.14 to −0.04)	−0.02 (−0.07 to 0.03)
By timing of exposure, trimester				
First	677	0.05 (1.0)	−0.15 (−0.23 to −0.08)	−0.06 (−0.13 to 0.02)
Second	800	0.15 (1.0)	−0.05 (−0.12 to 0.02)	0.01 (−0.06 to 0.08)
Third	492	0.13 (1.0)	−0.07 (−0.16 to 0.02)	0.00 (−0.08 to 0.09)
By duration of exposure, intervals^b^				
1	929	0.14 (1.0)	−0.06 (−0.13 to 0.00)	−0.01 (−0.07 to 0.06)
2-3	322	0.05 (1.0)	−0.16 (−0.27 to −0.05)	−0.11 (−0.22 to 0.01)
≥4	218	0.10 (1.0)	−0.10 (−0.23 to 0.02)	0.03 (−0.09 to 0.16)
**English**				
Unexposed	60 976	0.08 (1.0)	1 [Reference]	1 [Reference]
Exposed	1444	0.05 (1.0)	−0.03 (−0.08 to 0.02)	−0.01 (−0.07 to 0.04)
By timing of exposure, trimester				
First	672	0.01 (1.0)	−0.07 (−0.14 to 0.01)	−0.03 (−0.11 to 0.04)
Second	780	0.08 (1.0)	0.00 (−0.07 to 0.07)	0.00 (−0.07 to 0.08)
Third	484	0.08 (1.0)	0.00 (−0.09 to 0.09)	0.00 (−0.08 to 0.09)
By duration of exposure, intervals^b^				
1	917	0.05 (1.0)	−0.03 (−0.09 to 0.04)	−0.01 (−0.07 to 0.06)
2-3	313	0.01 (1.0)	−0.07 (−0.19 to 0.04)	−0.08 (−0.19 to 0.04)
≥4	214	0.11 (1.0)	0.03 (−0.10 to 0.16)	0.06 (−0.08 to 0.20)

^a^ β, standardized mean difference in test scores.

^b^ One interval corresponds to a 4-week period, but not necessarily consecutive use in that period.

## Discussion

To our knowledge, this study was the first to examine scholastic skills in children prenatally exposed to opioid analgesics. Our findings extended our understanding of the safety of prenatal opioid analgesic exposure in terms of neurodevelopment. In a large birth cohort, we found that children with any exposure to opioid analgesics during pregnancy showed scholastic scores similar to those of children of mothers with only prepregnancy exposure. However, exposure to opioid analgesics in the first trimester or during two to three 4-week intervals during pregnancy was associated with lower scores in literacy and numeracy, compared with only prepregnancy exposure. The differences in mean test scores were small; thus, they should be interpreted with caution.

The timing and duration of medications given during pregnancy are important in assessing safety.^38^ Organogenesis occurs in the first trimester, and the brain develops throughout the entire pregnancy.^39,40^ A potential explanation for our observation that exposure in the first trimester was associated with lower scholastic performance might be explained by immediate birth outcomes or specific malformations, which have been associated with a high risk of cognitive impairments.^41,42^ However, the literature is inconclusive regarding analgesic opioid use during pregnancy and the risk of malformations and/or immediate birth outcomes.^43,44,45,46,47,48^ Therefore, the observed association might not be attributable to the risk of adverse birth outcomes.

Two recent studies^10,11^ suggested that longer prenatal opioid analgesic exposures were associated with adverse neurodevelopmental outcomes. In the present study, we found that prenatal opioid analgesic exposures in two to three 4-week intervals were associated with low literacy and numeracy scores (wβ, −0.19; 95% CI, −0.35 to −0 04). However, exposure in 4 intervals or more was not associated with the scores. The exposed sample size was small; thus, results should be interpreted with caution. In our sensitivity analysis, when exposures in 2 or more intervals were compared with exposures in 1 interval only, we found no statistically significant difference in scholastic performance. This finding suggests that residual confounding or chance might have affected our primary analysis of exposure durations.

In the weighted subanalysis, with unexposed children as the reference, prenatal exposures to opioid analgesics in any trimester or for any duration were not associated with lower scores on tests in literacy, numeracy, or English. Moreover, in the main analysis, when we used only prepregnancy exposure as reference, we found greater differences in mean scores. This finding was somewhat counterintuitive, because we expected greater differences when unexposed children composed the reference group. However, this finding might be explained by the different weighting methods applied in the 2 analyses (inverse probability of treatment weights and standardized mortality and morbidity ratio weights), which may answer different questions (eMethods in the Supplement).^49^

The clinical relevance of our observations was difficult to evaluate owing to the lack of cutoff values for defining clinically significant differences. However, the observed differences were small.^50,51^ A standardized mean difference of −0.13 on literacy scores would correspond to an odds ratio of 1.3.^52^ Moreover, on all tests, the mean test scores among the exposed children were above the population mean, which indicated that their performance was not worse than that of the general population of children in the fifth grade. Taken together, our results suggest that prenatal exposure to opioid analgesics was not associated with poor fifth-grade scholastic skills. These findings may be useful for physicians advising pregnant women who need opioid analgesics for pain management. However, opioid analgesics are not the recommended first choice for treating pain during pregnancy. Although they may be used sporadically in the first and second trimesters,^53,54^ opioid analgesic use should be avoided in the third trimester owing to an increased risk of neonatal withdrawal symptoms.^55,56^

In Norway, national tests were introduced in 2007 as part of the national quality assessment system. Because they were not based on grades or teacher evaluations, the national tests were intended to provide an objective measure of scholastic skills to identify children who performed below the level of their peers.^24^ Scholastic skills reflect aspects of cognitive function,^17^ although the test results were not associated with IQ; instead, test results are a product of the child’s concentration, knowledge, and motivation for the given test.^15,19^ We decided to use the fifth-grade tests, because some disabilities are not detected until a child has problems in a school setting.^57^ It is essential to identify and help children with difficulties and put necessary measures in place, because problems with reading and writing are associated with a wide range of mental health problems, including anxiety, depression, and behavioral issues.^58^

We lack studies that examine neurodevelopmental outcomes in children after prenatal exposure to opioid analgesics.^9^ Most previous studies were conducted with women who used opioids for opioid maintenance therapy or for illicit purposes.^59,60^ However, those results are not generalizable to women who use opioid analgesics for pain management owing to differences in sociodemographic characteristics and lifestyle factors.^61,62^ Moreover, neurodevelopment includes a wide range of domains.^57^ Thus, further studies are needed to examine other domains of neurodevelopment.^9^

### Limitations

This study has limitations. Scholastic skills were measured at 1 time point and we only evaluated children in the fifth grade. We could not analyze the development of skills over time; therefore, it would be interesting to investigate performance in children at older ages. Reporting of opioid medication use is influenced by the accuracy of recall and willingness to report and may be subject to misclassification. Exposure misclassification is likely to be nondifferential because exposure data are collected before outcome assessment. This misclassification could have biased our results toward the null. We did not have information on opioid doses or durations in the MoBa cohort; thus, we used the number of 4-week intervals as a proxy for duration. A mother who had reported use of opioids during one 4-week interval may have used the drug only once or twice, not necessarily consecutively during that period. However, mothers who reported use during 2 or more 4-week periods are more likely to have consumed a higher total dose.^11^ The MoBa participation rate was 41%; thus, our cohort had a potential self-selection bias of the healthiest women.^63,64^ When interpreting our results, one should keep in mind that the generalizability may be limited to families that are above average in socioeconomic resources. We could not study the use of specific opioids at the substance level owing to the low number of children exposed per opioid substance. Future studies should endeavor to distinguish between specific opioids or strong and weak opioids, because they may be used for different indications.^65^ In addition, future studies should try to explore potential prenatal opioid treatment heterogeneity. We did not have information about use of illicit opioids during pregnancy and we cannot rule out residual confounding by the use of other medications, such as nonopioid analgesics, psychotropics, or triptans. On a national level, between 3% and 5% of Norwegian children are exempted from the national tests in each birth cohort.^24^ Some children may be exempted from 1 or 2 tests, but not necessarily all 3. Exemption was granted only for children in special education or special language training, and we cannot rule out that exemptions may have led to underestimations in the associations. In addition, we cannot rule out the potential of residual or unmeasured confounding on our results.

## Conclusions

Based on findings from a large Norwegian birth cohort, the scholastic skills of children in the fifth grade who had been exposed to opioid analgesics prenatally did not differ significantly from the skills of children in the fifth grade whose mothers had only prepregnancy exposures. These findings may be useful for physicians advising pregnant women as well as for pregnant women who need opioid analgesics for pain management. Adequate pain management in pregnancy should be discussed on an individual patient level, bearing in mind the benefits and risks of different analgesic therapies.

## Notes

Supplement. **eMethods.** Additional Methods **eResults.** Sensitivity Analyses **eTable 1.** Relevant Indications for Opioid Use During Pregnancy **eTable 2.** Test Scores Among Children in MoBa and All Children in Norway Who Took the Test, According to Subject and Test Year **eTable 3.** National Test Results of the MoBa Children Presented as *z* Scores **eTable 4.** Overview of Data Source of Covariates **eTable 5.** Characteristics of Generated Weights **eTable 6.** Overview of Alternative Model Specifications **eTable 7.** Use of Specific Opioids During Pregnancy **eTable 8.** Role of Gender on Scholastic Skills in Fifth Grade According to Exposure Status **eTable 9.** Association Between Duration of Prenatal Exposure to Opioid Analgesics and Scholastic Skills in Fifth Grade **eFigure 1.** Distribution of Test Scores in the Complete Population of Test Takers in Fifth Grade Between 2011 and 2018, According to Subject **eFigure 2.** Directed Acyclic Graph **eFigure 3.** Overview of Exempted Children and Their Exposure Status
