# Supplementary material for: Analysis of Prenatal Exposure to Opioid Analgesics and Scholastic Skills in Children in Fifth Grade in Norway
Source: JAMA Netw Open. 2022 Jul 19;5(7):e2222425. doi: 10.1001/jamanetworkopen.2022.22425 (PMC9297111; doi:10.1001/jamanetworkopen.2022.22425)
Supplement: Supplement. — eMethods. Additional Methods eResults. Sensitivity Analyses eTable 1. Relevant Indications for Opioid Use During Pregnancy eTable 2. Test Scores Among Children in MoBa and All Children in Norway Who Took the Test, According to Subject and Test Year eTable 3. National Test Results of the MoBa Children Presented as z Scores eTable 4. Overview of Data Source of Covariates eTable 5. Characteristics of Generated Weights eTable 6. Overview of Alternative Model Specifications eTable 7. Use of Specific Opioids During Pregnancy eTable 8. Role of Gender on Scholastic Skills in Fifth Grade According to Exposure Status eTable 9. Association Between Duration of Prenatal Exposure to Opioid Analgesics and Scholastic Skills in Fifth Grade eFigure 1. Distribution of Test Scores in the Complete Population of Test Takers in Fifth Grade Between 2011 and 2018, According to Subject eFigure 2. Directed Acyclic Graph eFigure 3. Overview of Exempted Children and Their Exposure Status [file jamanetwopen-e2222425-s001.pdf]

Supplementary Online Content

Trønnes JN, Lupattelli A, Ystrom E, Nordeng H. Analysis of prenatal exposure to opioid analgesics and scholastic skills in children in fifth grade in Norway. *JAMA Netw Open*. 2022;5(7):e2222425. doi:10.1001/jamanetworkopen.2022.22425

eMethods. Additional Methods

eResults. Sensitivity Analyses

eTable 1. Relevant Indications for Opioid Use During Pregnancy

eTable 2. Test Scores Among Children in MoBa and All Children in Norway Who Took the Test, According to Subject and Test Year

eTable 3. National Test Results of the MoBa Children Presented as *z* Scores

eTable 4. Overview of Data Source of Covariates

eTable 5. Characteristics of Generated Weights

eTable 6. Overview of Alternative Model Specifications

eTable 7. Use of Specific Opioids During Pregnancy

eTable 8. Role of Gender on Scholastic Skills in Fifth Grade According to Exposure Status

eTable 9. Association Between Duration of Prenatal Exposure to Opioid Analgesics and Scholastic Skills in Fifth Grade

eFigure 1. Distribution of Test Scores in the Complete Population of Test Takers in Fifth Grade Between 2011 and 2018, According to Subject

eFigure 2. Directed Acyclic Graph

eFigure 3. Overview of Exempted Children and Their Exposure Status

This supplementary material has been provided by the authors to give readers additional information about their work.

© 2022 Trønnes JN et al. *JAMA Network Open.*

eMethods. Additional Methods

Study sample:
The MoBa study recruited pregnant women between 1999-2008, however, they youngest children, born in the period 1999-2001, could not be included in the study due to lack of consent as they turned 18 before the end of follow- up in 2018.

Outcome:
National tests were introduced in Norway as part of the national quality assessment system in 2004, however, tests have been performed every year since 2007. National tests are mandatory, and only children with special educational or special language training needs were exempted from a test. National tests are held in the first semester of the fifth, eight, and ninth grade and test the basic skills of reading/literacy, numeracy, and English language. The pupils perform the tests on computers and have 90 minutes to complete one test. The tests are scored from zero and upwards.[1] eTable 2 and eTable 3 shows a simple comparison of scores between the MoBa participants and the complete population of test takers in each subject and each test year. eFigure 1 shows the distribution of scores in literacy, numeracy and English. Below is a more thorough description of what is being tested in each subject.

Literacy[1]
Reading and language is arguably the most important academic skill learned in school. The reading test is done by presenting the student with a text (roughly an A4 sheet 12pt text) and then asking multiple-choice questions in different complexity about the text. This measures speed of reading as well as the ability to decompose a longer segment of coherent text. The exams are designed to test for the following abilities: to find information in a text, to interpret a text, and to reflect and evaluate the content of a text. While volume training and exposure are important for most scholastic skills, this is especially important for reading. Volume training by reading different materials and books of increasing complexity is the primary way of excelling in reading and reflecting over writing content. The raw test scores range between a minimum of 0 points to a maximum of 34 points.

Numeracy[1]
Unlike reading, high volume and exposure is not sufficient for excelling in mathematics, with large volume being potentially detrimental to performance. The test should reflect multiple sub-domains in mathematics, such as numeracy, geometry, and statistics. Numeracy is the basics of number manipulation, but is limited to as addition, subtraction, multiplication, and division. Geometry and measurements, are the two and three dimensional representations of numeracy, where comparing volumes and surfaces of different measurements are evaluated. Statistics is operationalized as a visualization of data, mostly into charts and diagrams, with some data summary, such as mean, median, and factors. The raw test scores range between a minimum of 0 points to a maximum of 45 points.

English[1]
The tests are designed to measure three different aspects of English language; general reading, vocabulary and grammar. The raw test scores range between a minimum of 0 points to a maximum of 50 points.

Covariates:
Family income was assessed by income-to-needs-ratio (ITNR), which is family income after tax divided by the poverty level i.e. consumption equivalents. The EU-60 standard was applied.[2] The higher ITNR level, the better off are the family. ITNR was grouped into 3 levels (ITNR <2, ITNR 2-3, ITNR≥3). ITNR was assessed one year prior to childbirth.

Paternal medication use was collected in the Father's questionnaire. The fathers could report if they had used medications in the period 6 months before their partner became pregnant. Among our study sample of 64 256 children, we have data on 51 692 of the fathers. Among these, 943 (1.8 %) reported use of opioids. Among the fathers using opioids before their partner became pregnant (n=943), 5.0%, 3.7% and 91.3% had partners that used opioids during pregnancy, pre-pregnancy only and no use, respectively.

© 2022 Trønnes JN et al. *JAMA Network Open.*

Statistical analysis:
In a sub-analysis, we used unexposed children as reference group. For this analysis we used propensity scores with standardized mortality/morbidity ratio weights (SMR), instead of inverse probability of treatment (IPT) weights as were used in the main analysis with discontinuers. According to Stürmer et. al.[3] SMR weights are preferred when we have an unexposed reference group or when the reference group is not well defined. However, when we have an active reference (or disease comparator), the inverse probability of treatment (IPT) weights can be used. This is because with an active reference, we assume that the prevalence of the indication for treatment is somewhat similar between the two groups, thus reducing the potential for violations of the positivity assumption.[4] Then it would make sense to estimate the average treatment effect in the entire population and thus IPT weights can be used. IPT weights creates a pseudo-population of both the exposed and unexposed, which has the same covariate distribution as the overall population of exposed and unexposed. Every person is weighted by the inverse of the probability of receiving the treatment actually received. This answers the question: "what would have happened if everyone had been exposed versus what would have happened if everyone had not been exposed?" Standardized mortality/morbidity ratio (SMR) weights creates a pseudo-population of the unexposed, which has the same covariate distribution as the exposed. Every exposed person receives the weight of 1, while the unexposed are down-weighted and receives the weight of PS odds (PS/1-PS). With the SMR weights, we estimate the average treatment effect in the treated, which answers the question "what would have happened if those actually exposed had not been exposed?".

Subanalyses and sensitivity analyses:
In a sensitivity analysis, we compared children exposed to opioid analgesics in one period with children exposed in 2 or more intervals during pregnancy to examine associations with length of exposure.

In a sensitivity analysis, we used an alternative model specification (cf. eTable 6). Use of co-medications was measured in the period 6 months before pregnancy, and we removed symptoms of depression and anxiety measured in gestational week 17.

In the last sensitivity analysis, we restricted the study sample to women who had returned the MoBa questionnaire at 6 months post-partum (MoBa Q4), to ensure that every woman had complete information on exposure, and repeated our main analysis.

© 2022 Trønnes JN et al. *JAMA Network Open.*

eResults. Sensitivity Analyses

Children exposed to opioid analgesics in one interval scored similar to those exposed in two intervals or more during pregnancy (eTable 9).

In the analysis with the alternative model specification, point estimates did not differ substantially from the main analysis (data not shown). However, only associations of prenatal exposure in first trimester and in 2-3 4-week intervals with lower scores on the numeracy test remained significant. No other associations were found.

When MoBa Q4-returned was required to enter the study sample, the sample included 57 954 pregnancies. The point estimates were similar to those reported in the main analysis and conclusions remain the same (Data not shown).

© 2022 Trønnes JN et al. *JAMA Network Open.*

eTable 1. Relevant indications for opioid use during pregnancy.

Indications	MoBa Q1	MoBa Q3
Pelvic girdle pain	•	•
Abdominal pain	•	
Back pain	•	•
Neck and shoulder		
Arthritis	•	
Sciatica	•	
Fibromyalgia	•	
Other pains in muscles/joints		•
Migraine	•	
Other headache	•	
Headache / migraine		•

Abbrevations: MoBa, The Norwegian Mother, Father and Child Cohort; Q1, the first MoBa questionnaire; Q3, the third MoBa questionnaire.

© 2022 Trønnes JN et al. *JAMA Network Open.*

eTable 2. Test scores among children in MoBa and all children in Norway who took the test, according to subject and test year.

	Test year	MoBa children (n=93 416)		General population	
		N	Raw scores (mean ± SD)	N	Raw scores (mean ± SD)
Literacy	2011	14	24.0 ± 3.4	54 826	21.3 ± 5.9
	2012	7434	19.3 ± 6.0	54 319	18.6 ± 6.2
	2013	10 869	22.4 ± 6.5	55 314	21.5 ± 6.7
	2014	11 707	19.7 ± 5.8	55 862	18.8 ± 6.1
	2015	13 484	21.5 ± 6.3	55 611	20.3 ± 6.5
	2016	15 421	20.1 ± 7.0	58 297	18.4 ± 7.1
	2017	14 197	20.7 ± 6.1	58 192	19.0 ± 6.4
	2018	11 822	19.1 ± 5.9	59 792	17.4 ± 6.1
	Overall	84 948	20.5 ± 6.4	452 213	19.4 ± 6.5
Numeracy	2011	14	30.1 ± 6.5	55 122	26.0 ± 8.5
	2012	7510	27.7 ± 8.4	54 790	26.7 ± 8.6
	2013	11 063	26.5 ± 9.1	56 298	25.2 ± 9.4
	2014	11932	25.5 ± 9.1	57 235	24.1 ± 9.3
	2015	13 818	25.5 ± 9.0	57 117	23.8 ± 9.6
	2016	15 500	25.5 ± 9.0	58 829	23.3 ± 9.2
	2017	14 246	25.2 ± 9.3	58 710	22.8 ± 9.5
	2018	11 897	27.6 ± 9.1	60 478	24.8 ± 9.5
	Overall	85 980	26.1 ± 9.2	458 579	24.6 ± 9.3
English	2011*	-	-	-	-
	2012	7445	27.7 ± 11.3	54 426	27.3 ± 11.4
	2013	10 996	27.9 ± 10.5	55 978	27.5 ± 10.6
	2014	11 829	27.5 ± 9.8	56 764	26.8 ± 10.0
	2015	13 723	26.8 ± 10.4	56 625	26.3 ± 10.6
	2016	15 365	26.6 ± 9.3	58 229	25.7 ± 9.5
	2017	14 142	27.4 ± 9.6	58 080	26.4 ± 9.8
	2018	11 788	27.4 ± 10.4	59 795	26.4 ± 10.7
	Overall	85 288	27.3 ± 10.1	399 897	26.6 ± 10.4

Abbrevations: MoBa, The Norwegian Mother, Father and Child cohort

* Due to large technical problems the Norwegian directorate of education and training cancelled national tests in English in 2011.[5]

© 2022 Trønnes JN et al. *JAMA Network Open.*

eTable 3. National test results of the MoBa children presented as *z* scores.

	MoBa children, n=93 416		
**Test subject**	**N**	**Mean scores (SD)**	**Median scores (P25, P75)**
Literacy 5th grade	84 948	0.21 (0.97)	0.37 (-0.48, 0.97)
Numeracy 5th grade	85 980	0.20 (0.98)	0.23 (-0.54, 0.96)
English 5th grade	85 288	0.07 (0.98)	0.03 (-0.69, 0.88)

Abbrevations: MoBa, The Norwegian Mother, Father and Child cohort
P25 indicate the 25th percentile, and P75 indicate the 75th percentile of the z-score.

© 2022 Trønnes JN et al. *JAMA Network Open.*

eTable 4. Overview of data source of covariates.

Variable	Data Source
Maternal age	MBRN
Marital status	MoBa Q1
Parity	MBRN
Education level	SSB
Family income	SSB
Pre-pregnancy BMI	MoBa Q1
Smoking status at start of pregnancy	MBRN
Alcohol use	MoBa Q1
Symptoms of anxiety depression	MoBa Q1
Maternal chronic diseases before pregnancy	MoBa Q1
Co-medication<br>-Paracetamol<br>-Triptans<br>-NSAIDS<br>-Antidepressants<br>-Benzodiazepine and benzodiazepine-like drugs<br>-Antiepileptic<br>-Anti-psychotics	MoBa Q1
Paternal age	MBRN
Paternal education	SSB
Child sex	MBRN
Time of year the baby was born	MBRN

Abbrevations: MBRN, The Medical Birth Registry of Norway; MoBa, The Norwegian Mother, Father and Child Cohort; SSB, Statistics Norway; Q1, The first MoBa questionnaire.

© 2022 Trønnes JN et al. *JAMA Network Open.*

eTable 5. Characteristics of generated weights

	Literacy		Numeracy		English	
	Estimated IPTW		Estimated IPTW		Estimated IPTW	
	**Mean (SD)**	**Min-Max**	**Mean (SD)**	**Min-Max**	**Mean (SD)**	**Min-Max**
Pre-pregnancy exposed only	Reference	Reference	Reference	Reference	Reference	Reference
Exposed	1.0 (0.3)	0.6-5.6	1.0 (0.3)	0.6-5.3	1.0 (0.3)	0.6-5-8
**Timing of exposure**						
1st trimester	1.0 (0.5)	0.5-9.7	1.0 (0.6)	0.5-8.4	1.0 (0.5)	0.5-9.7
2nd trimester	1.0 (0.3)	0.5-4.8	1.0 (0.3)	0.5-5.6	1.0 (0.4)	0.5-6.4
3rd trimester	1.0 (0.4)	0.4-5.4	1.0 (0.4)	0.4-5.1	1.0 (0.4)	0.4-6.2
**Duration of exposure**						
1 interval	1.0 (0.2)	0.6-3.3	1.0 (0.2)	0.6-3.0	1.0 (0.2)	0.6-3.0
2-3 intervals	1.0 (0.4)	0.3-4.9	1.0 (0.4)	0.3-4.7	1.0 (0.4)	0.3-4.8
4 or more intervals	1.0 (0.6)	0.2-11.2	0.9 (0.7)	0.2-13.6	1.0 (0.7)	0.2-16.2

Abbreviations: IPTW, Inverse probability of treatment weights; SD, Standard deviation.

© 2022 Trønnes JN et al. *JAMA Network Open.*

eTable 6. Overview of alternative model specifications.

	Model	Description
Main	1	**sIPTW:**<br>**Numerator:**<br>Probability of exposure.<br>**Denominator:**<br>Maternal age, marital status, parity, maternal and paternal education level (in birth year), family income-to-needs-ratio (1 year prior to child birth), pre-pregnancy BMI, chronic disease pp, alcohol use (Q1), smoking habits at pregnancy start, use of co-medications (Q1), symptoms of anxiety and depression (Q1), time of year the baby was born (before/after summer) and paternal age.
Alternative model	2	**sIPTW:**<br>**Numerator:**<br>Probability of exposure.<br>**Denominator:**<br>Maternal age, marital status, parity, maternal and paternal education level (in birth year), family income-to-needs-ratio (1 year prior to child birth), pre-pregnancy BMI, chronic disease pp, smoking habits at pregnancy start, use of co-medications (pp), time of year the baby was born (before/after summer) and paternal age.<br><br>In this model, all covariates included are measured before pregnancy.

Abbreviations: sIPTW: stabilized inverse probability of treatment weight; BMI, body mass index; pp, pre-pregnancy; Q1, the first MoBa questionnaire. Symptoms of anxiety/depression was measured by a short version of the Hopkins Symptom Checklist (SCL-5) in the first MoBa questionnaire.

© 2022 Trønnes JN et al. *JAMA Network Open.*

eTable 7. Use of specific opioids during pregnancy.

Substance (ATC code)	Exposed during pregnancy, n=1483 (%)
Morphine (N02AA01)	68 (4.6)
Oxycodone (N02AA05)	9 (0.6)
Codeine, combinations excluding psycholeptics (N02AA59)	1342 (90.5)
Ketobemidone (N02AB01)	54 (3.6)
Buprenorphine (N02AE01)	3 (0.2)
Ketobemidone and antispasmodics (N02AG02)	18 (1.2)
Tramadol (N02AX02)	41 (2.8)

Women may have used more than one medication.
Abbreviations: ATC, Anatomical Therapeutic Chemical Classification system.

© 2022 Trønnes JN et al. *JAMA Network Open.*

eTable 8. Role of gender on scholastic skills in fifth grade according to exposure status.

		Boys			Girls		
		N	Crude β (95% CI)	Weighted β (95% CI)	N	Crude β (95% CI)	Weighted β (95% CI)
Literacy sample	Pre-pregnancy exposed	353	Reference	Reference	368	Reference	Reference
	Exposed	717	-0.05 (-0.18, 0.08)	-0.04 (-0.19, 0.11)	728	-0.07 (-0.18, 0.05)	-0.08 (-0.20, 0.04)
	Unexposed	30 608	Reference	Reference	30 101	Reference	Reference
	Exposed	717	-0.04 (-0.11, 0.04)	0.01 (-0.06, 0.09)	728	-0.07 (-0.14, -0.01)	-0.03 (-0.10, 0.04)
Numeracy sample							
	Pre-pregnancy exposed	354	Reference	Reference	368	Reference	Reference
	Exposed	731	-0.07 (-0.20, 0.05)	-0.06 (-0.19, 0.08)	738	-0.11 (-0.23, 0.002)	-0.10 (-0.22, 0.01)
	Unexposed	31 125	Reference	Reference	30 325	Reference	Reference
	Exposed	731	-0.04 (-0.12, 0.03)	0.02 (-0.05, 0.10)	738	-0.13 (-0.20, -0.06)	-0.06 (-0.13, 0.01)
English sample	Pre-pregnancy exposed	353	Reference	Reference	365	Reference	Reference
	Exposed	718	-0.003 (-0.14, 0.13)	-0.03 (-0.17, 0 12)	726	-0.04 (-0.16, 0.08)	-0.05 (-0.18, 0.07)
	Unexposed	30 765	Reference	Reference	30 211	Reference	Reference
	Exposed	718	0.02 (-0.06, 0.09)	0.04 (-0.04, 0.11)	726	-0.08 (-0.14, -0.01)	-0.06 (-0.13, 0.01)

β: indicates standardized mean difference in test scores.

© 2022 Trønnes JN et al. *JAMA Network Open.*

eTable 9. Association between duration of prenatal exposure to opioid analgesics and scholastic skills in fifth grade.

		Literacy		Numeracy		English	
	**N**	**Crude β (95% CI)**	**Weighted β (95% CI)**	**Crude β (95% CI)**	**Weighted β (95% CI)**	**Crude β (95% CI)**	**Weighted β (95% CI)**
**Exposed in 1 interval**	917	Reference	Reference	Reference	Reference	Reference	Reference
**Exposed in 2 or more intervals**	527	-0.08 (-0.19, 0.02)	-0.09 (-0.20, 0.02)	-0.07 (-0.18, 0.03)	-0.06 (-0.17, 0.05)	-0.01 (-0.11, 0.10)	-0.03 (-0.14, 0.09)

β: indicates standardized mean difference in test scores

© 2022 Trønnes JN et al. *JAMA Network Open.*

eFigure 1. Distribution of test scores in the complete population of test takers in fifth grade between 2011 and 2018, according to subject (A, B, C).

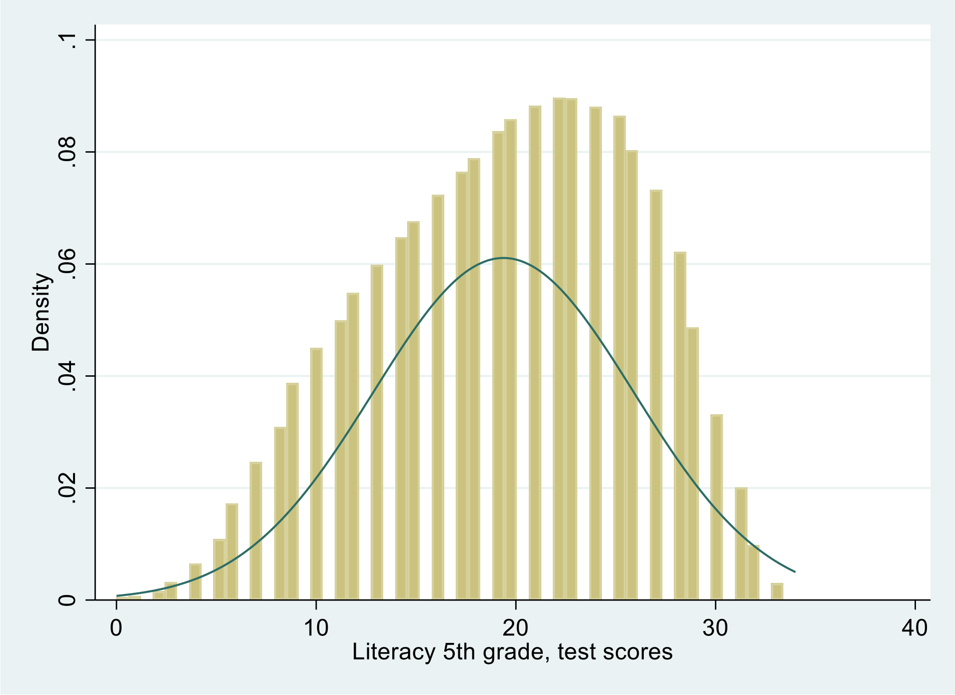

A) Test scores on the literacy test.
Mean (SD): 19.4 ± 6.5
Median (Range): 20 (0-34)

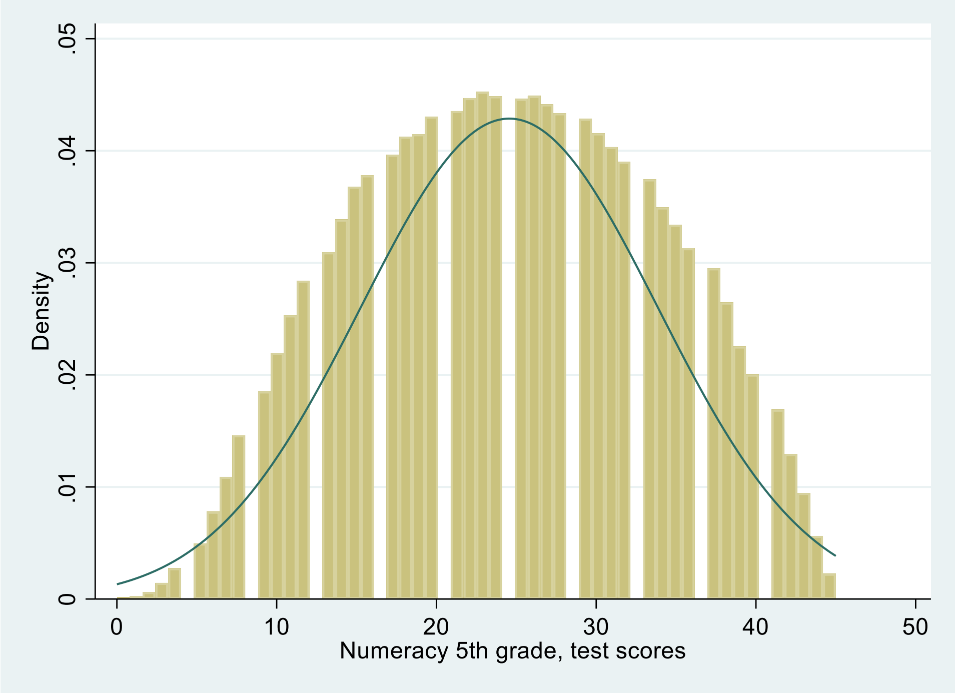

B) Test scores on the numeracy test
Mean (SD): 24.6 ± 9.3
Median (Range): 25 (0-45)

© 2022 Trønnes JN et al. *JAMA Network Open.*

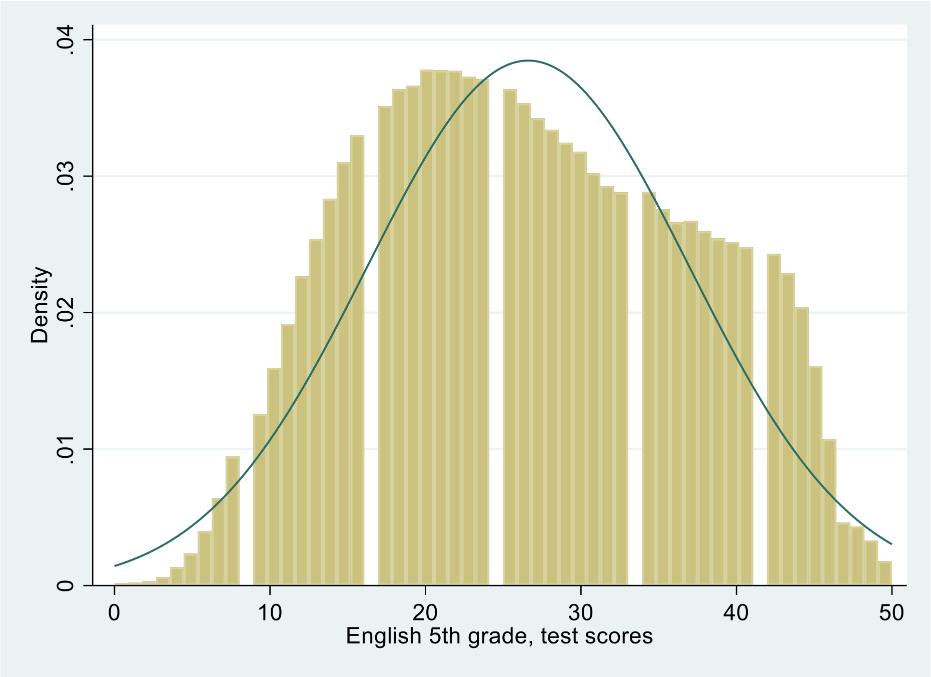

C) Test scores on the English test
Mean (SD): 26.6 ± 10.4
Median (Range): 26 (0-50)

© 2022 Trønnes JN et al. *JAMA Network Open.*

eFigure 2. Directed acyclic graph.

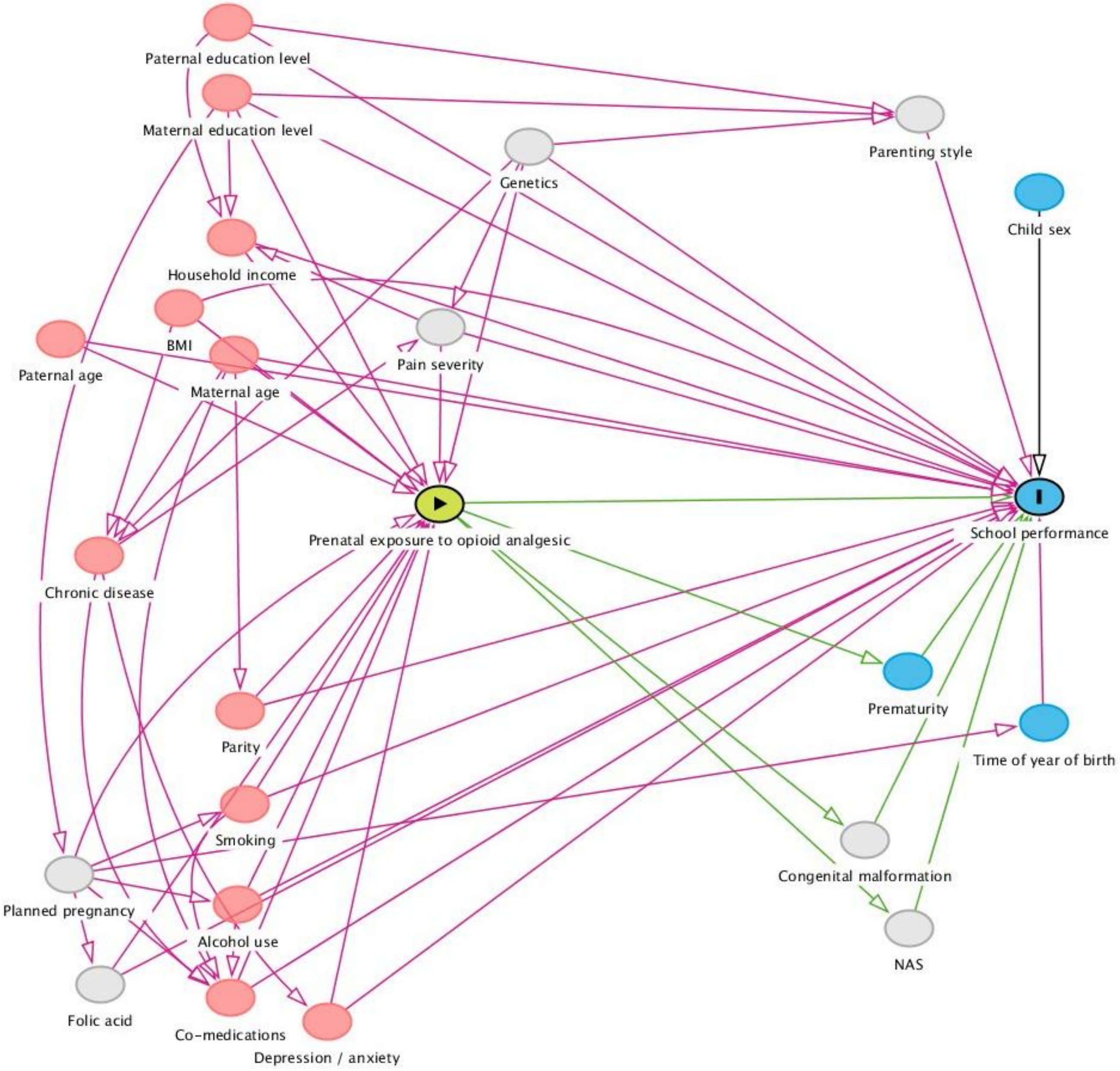

Simplified directed acyclic graph showing assumed relationship between the exposure (prenatal opioid analgesics), the outcome (school performance in children) and different covariates (e.g. sociodemographic and lifestyle factors, use of co-medications etc.).

Abbreviations: NAS, neonatal abstinence syndrome; BMI, body mass index.

© 2022 Trønnes JN et al. *JAMA Network Open.*

eFigure 3. Overview of exempted children and their exposure status.

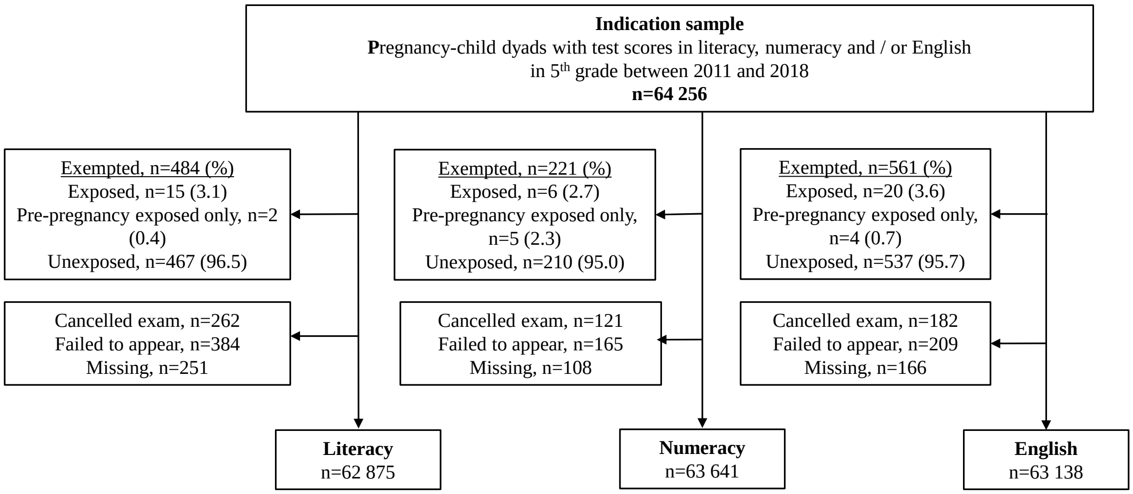

© 2022 Trønnes JN et al. *JAMA Network Open.*

eReferences

1. The Norwegian Directorate for Education and Training. Kva er nasjonale prøver? . https://www.udir.no/eksamen-og-prover/prover/nasjonale-prover/om-nasjonale-prover/. Accessed November 12, 2021.
2. Eurostat. Statistics Explained. Income to needs ratio. https://ec.europa.eu/eurostat/statistics-explained/index.php?title=Glossary:Equivalised_disposable_income. Accessed November 11, 2021.
3. Stürmer T, Wyss R, Glynn RJ, Brookhart MA. Propensity scores for confounder adjustment when assessing the effects of medical interventions using nonexperimental study designs. *J Intern Med.* 2014;275(6):570-580.
4. Hernán MA, Robins JM. Estimating causal effects from epidemiological data. *J Epidemiol Community Health.* 2006;60(7):578-586.
5. Engen TH. Nedenes skole. Nasjonale prøver i engelsk 5.trinn 2011. https://www.nedenes.skole.arendal.no/index.php?artID=344. Accessed November 10, 2021.

© 2022 Trønnes JN et al. *JAMA Network Open.*
